# MRI Assessment of Lung Water Density in Individuals Previously Infected With COVID‐19: A Cross‐Sectional Study

**DOI:** 10.1002/jmri.29814

**Published:** 2025-05-08

**Authors:** Christopher Keen, Justin Grenier, Peter Šereš, Robert Stobbe, James White, Christian Beaulieu, Rachel Sherrington, Amy Kirkham, D. Ian Paterson, Richard Thompson

**Affiliations:** ^1^ Department of Biomedical Engineering University of Alberta Edmonton Alberta Canada; ^2^ Department of Radiology and Diagnostic Imaging University of Alberta Edmonton Alberta Canada; ^3^ Libin Cardiovascular Institute University of Calgary Calgary Canada; ^4^ Faculty of Kinesiology & Physical Education University of Toronto Toronto Ontario Canada; ^5^ Division of Cardiology University of Ottawa Heart Institute Ottawa Ontario Canada

**Keywords:** COVID‐19, lung water density (LWD), UTE

## Abstract

**Background:**

Lung damage in post‐acute COVID‐19 is a common clinical finding. Lung water density (LWD) imaging using ultrashort echo time (UTE) MRI with proton‐density weighting is sensitive to edema and fibrosis.

**Purpose:**

To characterize LWD in COVID‐19 survivors, compared with a healthy cohort.

**Study Type:**

Retrospective cohort.

**Populations:**

185 COVID‐19 survivors (63 male; age [median (interquartile range, IQR)]: 51 (25–83) years; 160 (66–363) days from COVID‐19 infection to MRI) and 109 healthy controls (64 male; age: 52 (27–76) years) with no history of COVID‐19 infection.

**Field Strength/Sequence:**

2.89T; Yarnball UTE pulse sequence.

**Assessment:**

Free‐breathing three‐dimensional LWD images were acquired in both cohorts. Clinical demographics (age, sex, body mass index [BMI]), presence of comorbidities (hypertension, dyslipidemia, diabetes, obesity), COVID‐19 hospitalization, pulmonary function, six‐minute walking distance, and plasma biomarkers were recorded.

**Statistical Tests:**

Student's *t*‐tests or Mann–Whitney *U* tests were used to compare lung water metrics between cohorts. The effect of comorbidities was assessed using Kruskal–Wallis tests followed by pairwise Wilcoxon tests with Bonferroni correction. Categorical variables were compared using chi‐squared tests. *p* < 0.05 was considered significant.

**Results:**

LWD (median (IQR)), was significantly greater in the post‐COVID‐19 cohort than in the healthy cohort, 31.3 (6.6)% versus 27.9 (6.5)% in men and 30.3 (7.4)% versus 27.5 (4.9)% in women. 37% of men and 24% of women in the post‐COVID‐19 cohort had LWD above the healthy cohort 95% confidence limit. Participants with elevated LWD had significantly higher BMI (kg/m^2^) (32 (5) versus 26 (4) in men, 33 (9) versus 26 (7) in women), incidence of comorbidities (78% vs. 50% in men, 72% vs. 38% in women), rates of COVID‐19 hospitalization (52% vs. 23% in men, 38% vs. 18% in women), and elevated CRP (mg/L) (2.2 (3.4) vs. 1.1 (1.4) in men, 1.8 (4.2) vs. 1.2 (2.1) in women).

**Data Conclusion:**

MRI‐derived LWD is elevated in COVID‐19 survivors and is related to high BMI, COVID‐19 hospitalization, inflammatory plasma biomarkers, and the presence of comorbidities.

**Evidence Level:**

2.

**Technical Efficacy:**

Stage 3.

**Plain Language Summary**

- We evaluated differences in lung water density (LWD) following recovery from COVID‐19 infection compared with those with no history of COVID‐19 infection.
- LWD, measured with MRI, provides a measure of the amount of water in the lungs, which can be increased with tissue damage.
- We found that LWD was increased following recovery from COVID‐19 infection.
- We also found that factors such as obesity, diabetes, and hospitalization for COVID‐19 infection were associated with higher LWD values.

## 1 Introduction

Since the start of the global COVID‐19 pandemic in late 2019, over 700 million people have been infected with the SARS‐CoV‐2 virus [1]. The long‐term effects of COVID‐19 have been widely reported and are broad in scope, affecting an estimated 10% of infected individuals; however, their underlying mechanisms and effective treatments remain unknown [2]. Pulmonary symptoms and lung imaging findings are among the most common during both the acute and early post‐acute phases of COVID‐19 infection [2, 3, 4]. Lung computed tomography (CT) studies > 2 months after COVID‐19 recovery have shown diminishment of the ground‐glass opacities common in acute studies and a greater incidence of fibrosis‐like features [5, 6, 7, 8]. Air trapping has also been observed in post‐acute lung CT images [9, 10]. Together, these findings point to fibrotic injury, microthrombus formation, and persistent inflammation as potential underlying mechanisms for pulmonary symptoms and radiographic findings [2, 4, 8, 11].

Despite the success of CT in identifying structural lung abnormalities in COVID‐19 survivors, the information provided is largely qualitative (e.g., appearance of ground glass opacities) and requires exposure to ionizing radiation. MRI is an alternative imaging modality for the clinical evaluation of the lungs, especially when serial imaging is required [12, 13] and custom MRI methods can provide features similar to those of lung CT scans without exposure to ionizing radiation [13, 14]. In particular, quantitative lung water density (LWD) MRI combines ultrashort echo time (UTE) proton density weighted MRI with spatial normalization to yield images of LWD [15, 16]. By comparison, increased Hounsfield units in CT images of the lungs reflect an unknown combination of increased water content and solid tissues (e.g., fibrotic tissue). MRI‐derived LWD has been shown to be sensitive to subclinical pulmonary edema and to have prognostic value in heart failure patients [15, 17, 18]. LWD has also been used as a biomarker in asthma and cystic fibrosis cohorts [19, 20] and is sensitive to gravity and exercise‐stress–induced changes to lung water distribution [21, 22, 23]. However, LWD measurements in COVID‐19 survivors are scarce.

Thus the aim of this cross‐sectional study was to characterize LWD following recovery from COVID‐19 infection, with comparison to a healthy cohort with no history of COVID‐19 infection. Further aims were to investigate the associations between LWD and clinical demographics, presence of comorbidities, pulmonary function, exercise capacity, and plasma biomarkers in the COVID‐19 cohort.

## 2 Methods

### 2.1 Study Participants and Clinical Evaluation

This study was approved by the local Health Research Ethics Boards and written informed consent was obtained from all study participants. 185 COVID‐19 survivors (post‐COVID‐19) with proof of PCR test results and 109 healthy controls without a history of any diagnosed diseases, conditions requiring regular medication, or prior COVID‐19 infection were enrolled. All participants were required to be over 18 years of age, and post‐COVID‐19 participants needed to have proof of infection no less than 12 weeks prior to MRI. Participant demographics are summarized in Table 1. In addition to lung MRI studies, all participants completed a six‐minute walking distance (6MWD) test and a pulmonary function test (PFT), which included 1 s forced expiratory volume (FEV_1_) and forced vital capacity (FVC). Post‐COVID‐19 participants also had blood drawn for lab analysis of B‐type natriuretic peptide (BNP), C‐reactive protein (CRP), lactate dehydrogenase (LDH), D‐dimer, and white cell count (WCC). LWD was compared between controls and post‐COVID‐19 participants with consideration of the presence or absence of comorbidities, including one or more of hypertension, dyslipidemia, diabetes (type 1 or type 2), or obesity (BMI > 30). MRI studies, clinical evaluations, and blood draws were completed on the same day.

**TABLE 1:** Participant demographic information at time of MRI.

	Healthy (*n* = 109)	Post‐COVID‐19 (*n* = 185)	*p*
Age, years (range)	52 (27–76)	51 (25–83)	0.700
No. of men, *n* (%)	54 (46)	63 (22)	0.010
BMI, kg/m^2^	24 (4)	28 (7)	< 0.001
Height (men), *m*	177 (9)	175 (9)	0.600
Height (women), *m*	164 (8)	164 (11)	0.862
Weight (men), kg	77.4 (16.1)	87.0 (13.6)	< 0.001
Weight (women), kg	63.2 (11.8)	74.0 (25.8)	< 0.001
SpO_2_, %	97 (2)	98 (2)	0.400
Heart rate, beats/min	70 (13)	73 (15)	0.060
Systolic BP, mmHg	121 (18)	130 (25)	< 0.001
Diastolic BP, mmHg	80 (13)	81 (14)	0.080
Time of MRI from COVID‐19 diagnosis, days (range)	—	160 (66–363)	—
Hospitalized, *n* (%)	—	49 (26)	—
Duration of hospital stay, days (range)	—	8 (1–91)	—
Current smoker, *n* (%)	1 (1)	7 (4)	0.300
COPD, *n* (%)	0 (0)	20 (11)	< 0.001
Hypertension, *n* (%)	0 (0)	40 (22)	< 0.001
Dyslipidemia, *n* (%)	0 (0)	32 (17)	< 0.001
Type 1 diabetes, *n* (%)	0 (0)	2 (1)	0.462
Type 2 diabetes, *n* (%)	0 (0)	22 (12)	< 0.001
6MWD, *m*	624 (89)	572 (112)	< 0.001
FEV_1_, %_pred_	103 (16)	98 (19)	< 0.01
FVC, %_pred_	107 (13)	99 (18)	< 0.001
FEV_1_/FVC, %	77 (8)	80 (10)	< 0.001
BNP, pg/mL	—	36.0 (27.5)	—
CRP, mg/dL	—	1.4 (2.8)	—
LDH, U/L	—	153 (37)	—
D‐dimer, μg/mL	—	0.33 (0.22)	—
WCC, 10^3^/μL	—	6.6 (2.8)	—

*Note*: Data are presented as median (IQR) unless otherwise specified.

Abbreviations: 6MWD, six‐minute walking distance; BMI, body mass index; BNP, brain natriuretic peptide; COPD, chronic obstructive pulmonary disorder; CRP, C‐reactive protein; FEV_1_, forced expiration volume in 1 s; FVC, forced vital capacity; LDH, lactate dehydrogenase; SpO_2_, oxygen saturation; WCC, white blood cell count.

### 2.2 Image Acquisition and Reconstruction

Imaging studies at two sites were completed on 2.89T MRI systems (Prisma, Siemens, Erlangen, Germany) with spine and body coil arrays for signal reception (total of 36 coil elements). UTE proton density weighted images were acquired using a custom free‐breathing three‐dimensional (3D) pulse sequence with Yarnball k‐space sampling [16, 24]. LWD imaging with the Yarnball sequence has been previously described and validated [16].

#### 2.2.1 Acquisition Parameters

Yarnball Variant 1–300‐mm field of view (FOV) in all directions, echo time (*T*_*E*_) = 0.07 ms, repetition time (*T*_*R*_) = 1.97 ms, flip angle = 1°, 50° radiofrequency pulse phase increment for spoiling, readout duration (*T*_RO_) = 1.5 ms, voxel size = 2.5 mm isotropic (interpolated with zero padding to 2 mm during reconstruction), with a total acquisition time of 4 min. Yarnball Variant 2–350 mm FOV in all directions, *T*_*E*_ = 0.11 ms, *T*_*R*_ = 2.88 ms, flip angle = 1°, 50° radiofrequency pulse phase increment for spoiling, *T*_RO_ = 1.3 ms, voxel size = 3.5 mm isotropic (interpolated with zero padding to 2.2 mm during reconstruction), with a total acquisition time of 2 min. Variant 1 was used for the first 109 post‐COVID‐19 participants, whereas variant 2 was used for the final 76 post‐COVID‐19 participants and all healthy controls. A subset of 39 post‐COVID‐19 participants were imaged with both pulse sequence variants, acquired sequentially, to evaluate potential bias in LWD values with the adjustment of pulse sequence parameters.

All images were acquired during restful tidal breathing and reconstructed at functional residual capacity ([FRC], minimum lung volume over the respiratory cycle). A subset of k‐space trajectories at FRC was selected using a center of k‐space navigator to track the phase of lung inflation [16, 25]. k‐space trajectories were incremented in a golden‐ratio pattern to ensure an even and pseudorandom distribution throughout k‐space independent of breathing patterns. The final set of k‐space data from the targeted FRC respiratory phase was sampling‐density compensated to a beta = 2 Kaiser filtering shape and zero padded for interpolation to the final image resolution [26]. Finally, the data were gridded [27] and coil element datasets were combined using a modified sum of squares approach (SUPER) after Fourier transformation to image space [28].

### 2.3 Image Processing and Lung Water Quantification

**FIGURE 1:**
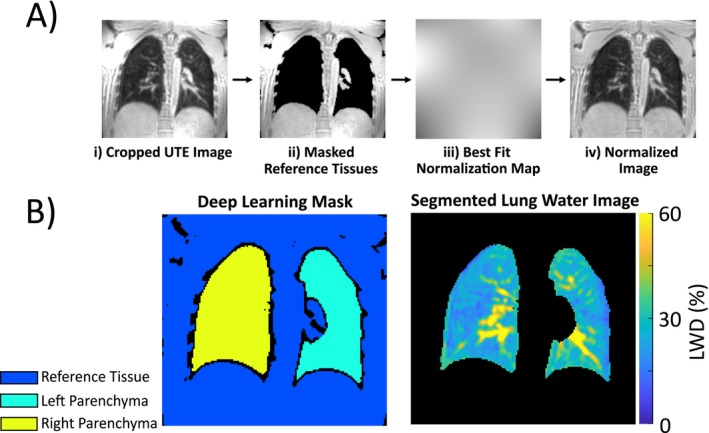
Overview of the lung water density quantification process: (A) Lung water density (LWD) normalization pipeline: (i) a cropped UTE image, (ii) deep learning identification of reference tissue, (iii) calculation of the normalization map, and (iv) application of the normalization map. (B) The corresponding segmented image and LWD map in the lung parenchyma. One of eighty‐eight coronal slices covering the full lung parenchyma is shown.

In the first step of the image processing pipeline, the images were cropped to exclude the arms, neck, and coronal slices outside of the body at the chest and back, with typical cropping shown in Figure 1Ai. A nnU‐Net deep learning model, previously trained on data from multiple UTE pulse sequences and validated on post‐COVID‐19 data, was then used to segment the left and right lung parenchyma and a separate segmented region including the surrounding solid tissues (skeletal muscle, fat, myocardium, blood, henceforth referred to as reference tissues) [29]. The nnU‐Net model is available for download at https://zenodo.org/records/15215411. A previously reported spatial normalization scheme was applied to simultaneously eliminate surface coil shading and to convert voxel intensity to units of relative LWD [16]. Briefly, the reference tissues surrounding the lungs were fit with a low spatial frequency normalization map that was interpolated over the full FOV using least square linear regression with Tikhonov regularization (Figure 1Aii,Aiii). Images were then corrected by division with the normalization map to yield voxel intensity in units of relative LWD (i.e., all signal intensities are relative to the surrounding solid tissues as a percentage) (Figure 1Aiv). Figure 1B depicts an example segmentation mask used for LWD processing as well as the resulting LWD map.

Whole‐LWD was calculated as the mean LWD value from all pixels within the lung parenchyma, identified using the deep‐learning‐generated masks for each lung. The total lung volumes were calculated using this same masked region. Additionally, lung water volume in both lungs was calculated as the sum of water volumes from all voxels within the masked regions, where the individual voxel water volume equals the voxel volume multiplied by the LWD voxel value.

Elevated LWD was defined as values above the upper limit of the 95% confidence interval (mean + 1.96 standard deviations) for the healthy cohort.

### 2.4 Statistical Methods

Shapiro–Wilks testing was used to assess continuous variables for normality. Depending on the results, Student's *t*‐test or Mann–Whitney *U* tests were used to compare lung water metrics, PFT findings, and 6MWD measurements between the healthy and post‐COVID‐19 cohorts. Additional comparisons within the post‐COVID‐19 cohort were conducted for blood biomarkers. Comparison of controls and post‐COVID‐19 groups with and without comorbidities used Kruskal–Wallis tests followed by pairwise Wilcoxon comparison with Bonferroni *p* value correction. Additionally, Spearman correlation was used to examine the relationship between BMI and LWD. Categorical variables such as prior hospitalization were compared using chi‐squared tests. Bland–Altman and correlation analysis were used to compare LWD measurements in post‐COVID‐19 participants imaged with both Yarnball pulse sequence variants. All statistical analysis was performed using R 4.2.3 (R Foundation for Statistical Computing, Vienna, Austria). A *p* value less than 0.05 was considered statistically significant.

## 3 Results

Post‐COVID‐19 and control cohorts were of similar age (*p* = 0.70) but with a significantly higher proportion of women in the patient group. The post‐COVID‐19 group was studied 160 days after COVID‐19 infection, and this group had significantly higher BMI, systolic blood pressure, and presence of other comorbidities (COPD, dyslipidemia, and diabetes) (Table 1).

### 3.1 Qualitative MRI Findings

**FIGURE 2:**
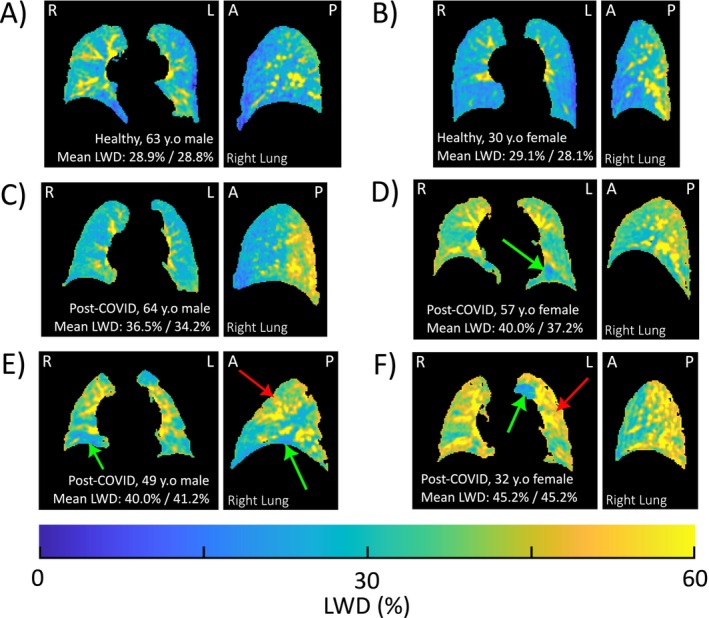
Representative lung water density images from healthy and post‐COVID‐19 participants: Coronal and sagittal (right lung) views of lung water density (LWD) images for two healthy subjects (A, B) and four cases from the post‐COVID‐19 cohort (C–F) with different global and regional increases in LWD. Red arrows indicate regions of patchy elevations in water density, and green arrows indicate regions of low water density. A, anterior; L, left; P, posterior; R, right. Mean LWD values indicated are for the right lung and left lungs.

**FIGURE 3:**
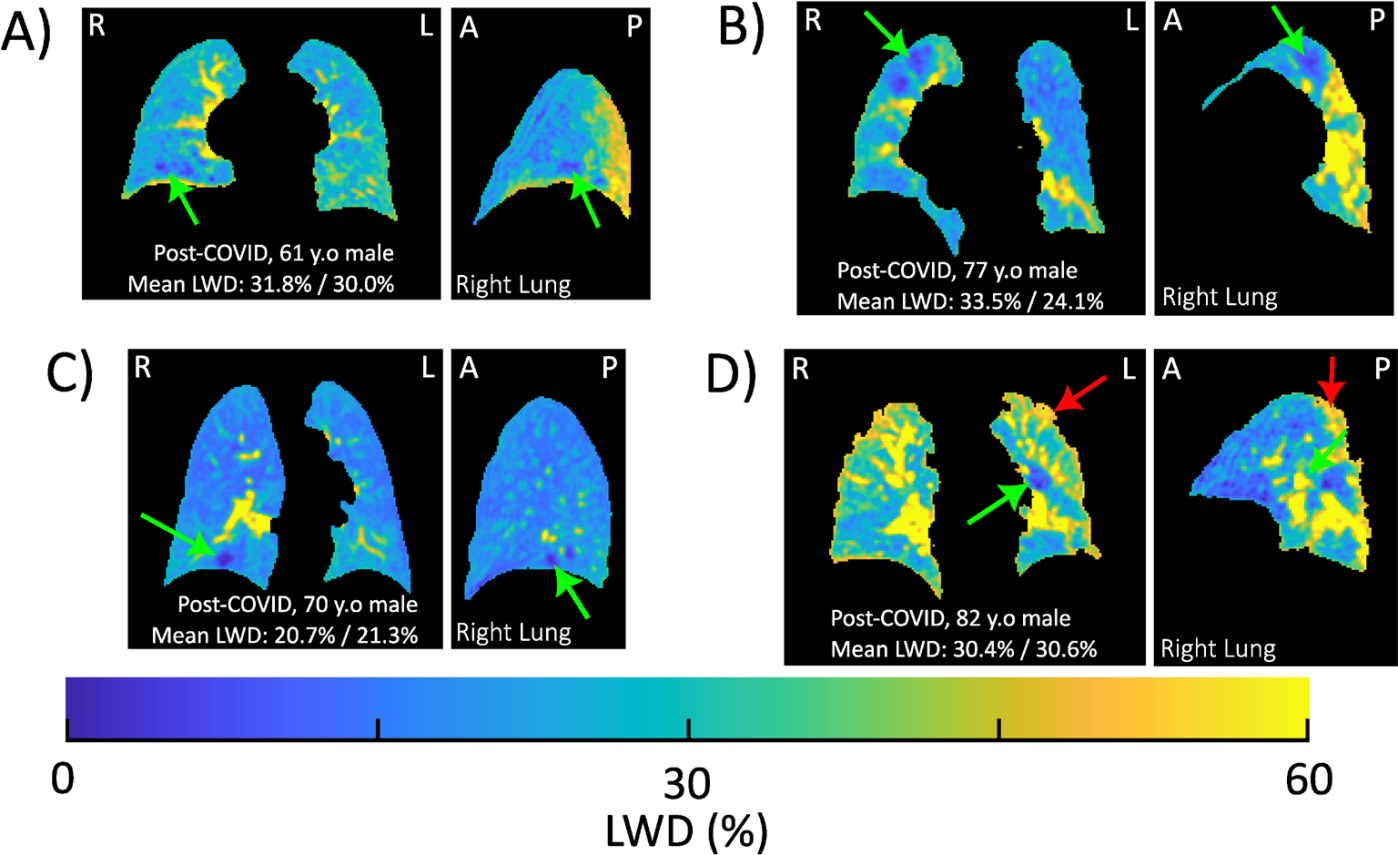
Lung water density images showing spatial nonuniformity in post‐COVID‐19 participants with normal lung water density: Coronal and sagittal views of lung water density (LWD) images for four cases from the post‐COVID‐19 cohort with normal global LWD values but patches of spatially non‐uniform LWD. Red arrows indicate regions of patchy elevations in water density, and green arrows indicate regions of low water density. A, anterior; L, left; P, posterior; R, right. Mean LWD values indicated are for the right lung and left lungs.

Lung water images in the post‐COVID‐19 cohort varied widely in appearance, commonly with patchy signal variations and with elevated water densities as compared with healthy controls (Figure 2). The presence of focal LWD elevations or dropout was also observed in post‐COVID‐19 participants with otherwise normal LWD as compared with controls, measured globally (Figure 3).

### 3.2 Quantitative Lung Metrics

Global lung water metrics for the healthy and post‐COVID‐19 cohorts are summarized in Table 2. Mean LWD was significantly higher in both lungs in the post‐COVID‐19 cohort as compared with the healthy cohort. Combined right and left LWD values (median (IQR)) were 27.9% (6.5%) in men and 27.5% (4.9%) in women in the healthy cohort versus 31.3% (6.6%) in men and 30.3% (7.4%) in women in the post‐COVID‐19 cohort. However, total water volume (mL) in the lungs was not significantly different between healthy and post‐COVID‐19 cohorts (*p* = 0.984/0.089 for the left/right lungs of men and *p* = 0.897/0.837 for women). Finally, lung volumes were significantly smaller in the right lung of the post‐COVID‐19 cohort as compared with healthy lungs, independent of sex, with a similar but nonsignificant trend in the left lungs (*p* = 0.073 for men and *p* = 0.087 for women).

**TABLE 2:** Lung water metrics in men and women in the healthy and post‐COVID‐19 cohorts.

	Men						Women					
	Left lung			Right lung			Left lung			Right lung		
	Healthy	Post‐COVID‐19	*p*	Healthy	Post‐COVID‐19	*p*	Healthy	Post‐COVID‐19	*p*	Healthy	Post‐COVID‐19	*p*
Median LWD (%)	28.8 (6.4)	32.0 (7.7)	0.002	26.8 (6.3)	31.8 (6.9)*	< 0.001	28.7 (4.4)	30.4 (7.9)	0.001	26.9 (5.3)	30.2 (6.9)*	< 0.001
Lung volume (mL)	1052 (339)*	926 (393)*	0.073	1344 (530)*	1114 (466)*	0.008	846 (232)*	787 (232)*	0.087	1065 (255)*	994 (291)*	0.007
Water volume (mL)	289 (96)*	291 (73)*	0.984	373 (87)*	378 (85)*	0.089	240 (42)*	239 (50)*	0.897	295 (48)*	295 (63)*	0.837

*Note*: Data are presented as median (IQR).

Abbreviation: LWD, lung water density.

* *p* < 0.05 for male–female comparison in a given cohort.

### 3.3 Age and Sex Dependence of LWD

**FIGURE 4:**
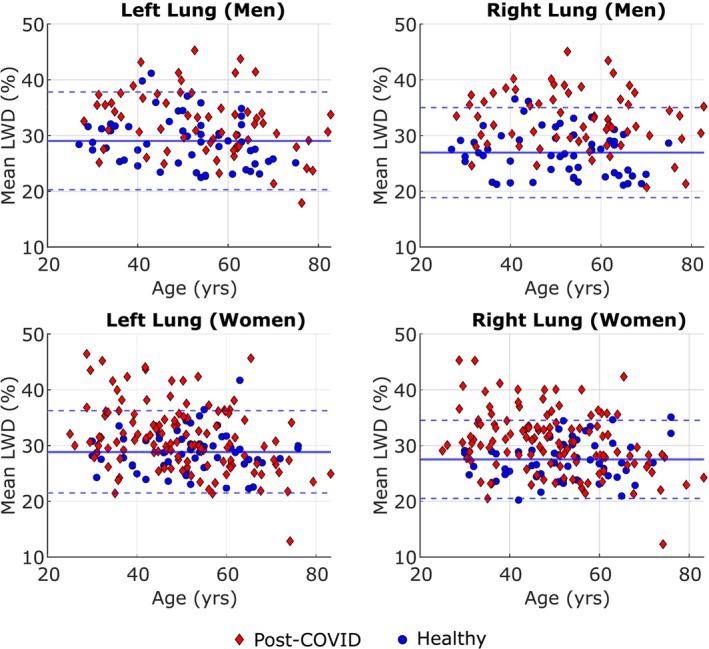
Age and sex variations in lung water density depicting the presence of elevated mean lung water density in the post‐COVID cohort: Mean lung water density (LWD) in men and women plotted against age in each lung for healthy and post‐COVID‐19 cohorts. The solid lines represent the healthy mean values and dotted lines indicate the 95% healthy confidence intervals (mean ± 1.96 standard deviations).

LWD was not significantly different between men and women in the healthy cohort (*p* = 0.832 for the left lung and *p* = 0.365 for the right lung), but was significantly higher in the right lung of men in the post‐COVID‐19 cohort. Lung volume and water volume were significantly higher in men than women in both cohorts. LWD was found to be negatively correlated with age in the post‐COVID‐19 cohort, whereas the healthy cohort showed no significance in either sex (*p* = 0.065/0.158 for the left/right lungs of men and *p* = 0.775/0.186 for women). The age and sex dependencies of LWD values for both lungs for all healthy and post‐COVID‐19 participants are illustrated in Figure 4.

### 3.4 LWD and Comorbidities

**FIGURE 5:**
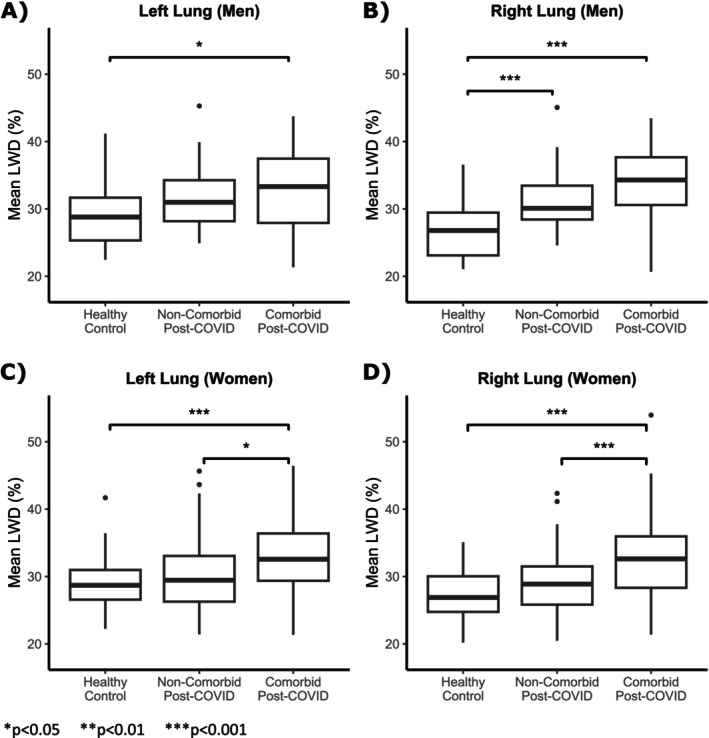
Lung water density comorbidity comparisons: Boxplots compare the mean lung water density (LWD) in healthy controls and post‐COVID‐19 participants, separately for the left and right lungs in men and women, with and without comorbidities (one or more of hypertension, dyslipidemia, diabetes [type 1 or type 2], or obesity [BMI > 30]).

Comparison of controls and post‐COVID‐19 subgroups with and without comorbidities revealed significant differences in LWD values between healthy participants and post COVID‐19 subgroups (Figure 5). Subsequent multiple comparison analysis showed that COVID‐19 survivors with comorbidities had significantly higher LWD in both lungs as compared with controls. LWD differences between controls and survivors without comorbidities were only significant in right lungs for men. Mean LWD was moderately correlated with BMI in the left and right lungs in men (*R* = 0.41/0.50 healthy and *R* = 0.38/0.52 post‐COVID) and women (*R* = 0.20/0.51 healthy and *R* = 0.45/0.50 post‐COVID), but with no significant relationship to height (*p* = 0.600 for men and 0.862 for women).

### 3.5 Associations With Elevated LWD

The subset of the post‐COVID‐19 with elevated LWD, with mean LWD values above the upper limit of the 95% healthy confidence interval (mean + 1.96 standard deviations) [15], are indicated in Figure 4. Twenty‐three men (37%) and 29 women (24%) were found to have elevated mean LWD in one or both lungs. Most women with elevated LWD were found to be under 50 years of age, whereas the ages of men with elevated LWD were more uniformly distributed over the full range of participants.

In general, both men and women with elevated LWD in one or both lungs had significantly greater BMI, significantly higher FEV_1_/FVC, significantly higher white blood cell counts, and were significantly more likely to have been hospitalized during the acute stage of infection and to have one or more of the identified comorbidities (Table 3). FEV_1_/FVC was significantly positively correlated with LWD in both men (*R* = 0.43) and women (*R* = 0.36).

**TABLE 3:** Comparison of post‐COVID participants with normal LWD and elevated LWD.

	Men			Women		
	Normal LWD (*N* = 40)	Elevated LWD (*N* = 23)	*p*	Normal LWD (*N* = 93)	Elevated LWD (*N* = 29)	*p*
Age	58 (19)	50 (21)	0.182	49 (20)	48 (22)	0.152
BMI, kg/m^2^	26 (4)	32 (5)	< 0.001	26 (7)	33 (9)	< 0.001
Mean LWD (%), left/right maximum	31 (5)	38 (3)	< 0.001	30 (5)	38 (6)	< 0.001
Mean LWD (%), left/right average	30 (4)	37 (3)	< 0.001	29 (5)	38 (6)	< 0.001
Time from COVID‐19 diagnosis to MRI, days	166 (63)	160 (83)	0.949	157 (78)	146 (69)	0.915
Hospitalized, *n* (%)	9 (23)	12 (52)	0.016	17 (18)	11 (38)	0.028
Duration of hospital stay, days	11 (15)	11 (9)	0.846	7 (5)	8 (7)	0.272
Comorbidity, *n* (%)	20 (50)	18 (78)	0.027	35 (38)	21 (72)	< 0.001
6MWD, *m*	582 (134)	575 (99)	0.792	577 (100)	528 (115)	0.034
FEV_1_, %_pred_	98 (24)	99 (13)	0.481	99 (21)	98 (14)	0.512
FVC, %_pred_	102 (25)	97 (10)	0.080	99 (19)	97 (15)	0.112
FEV_1_/FVC, %	78 (10)	82 (10)	0.007	80 (11)	84 (7)	0.014
BNP, pg/mL	49.0 (23.8)	27.0 (25.5)	0.068	41.0 (32.0)	28.0 (22.0)	0.040
CRP, mg/L	1.1 (1.4)	2.2 (3.4)	0.083	1.2 (2.1)	1.8 (4.2)	0.037
CRP ≥ 3 mg/L, *n* (%)	6 (15)	8 (35)	0.069	22 (24)	14 (48)	0.011
LDH, U/L	159 (32)	158 (41)	0.522	153 (33)	147 (49)	0.687
D‐dimer, μg/mL	0.38 (0.20)	0.29 (0.08)	0.126	0.33 (0.24)	0.36 (0.21)	0.542
WCC, 10^3^/μL	6.4 (2.3)	7.5 (2.2)	0.030	6.1 (2.5)	7.4 (2.6)	0.045

*Note*: Data are presented as median (IQR) unless otherwise specified.

Abbreviations: 6MWD, six‐minute walking distance; BMI, body mass index; BNP, brain natriuretic peptide; CRP, C‐reactive protein; FEV_1_, forced expiration volume in 1 s; FVC, forced vital capacity; LDH, lactate dehydrogenase; WCC, white blood cell count.

In women with elevated LWD, 6MWD was significantly shorter, and CRP levels were significantly greater, with a significantly higher fraction of participants with elevated LWD also having clinically elevated CRP levels above 3 mg/L. BNP levels were significantly lower in women in the elevated LWD group.

Participant age (*p* = 0.182 for men and *p* = 0.152 for women), time since COVID‐19 diagnosis (*p* = 0.949 for men and *p* = 0.915 for women), and (where applicable) duration of hospital stay (*p* = 0.846 for men and *p* = 0.272 for women) were not significantly associated with LWD elevation. Median FEV_1_, FVC, FEV_1_/FVC, WCC, BNP, LDH, and D‐dimer measurements were within the normal clinical range in post‐COVID‐19 participants with and without elevated LWD.

**FIGURE 6:**
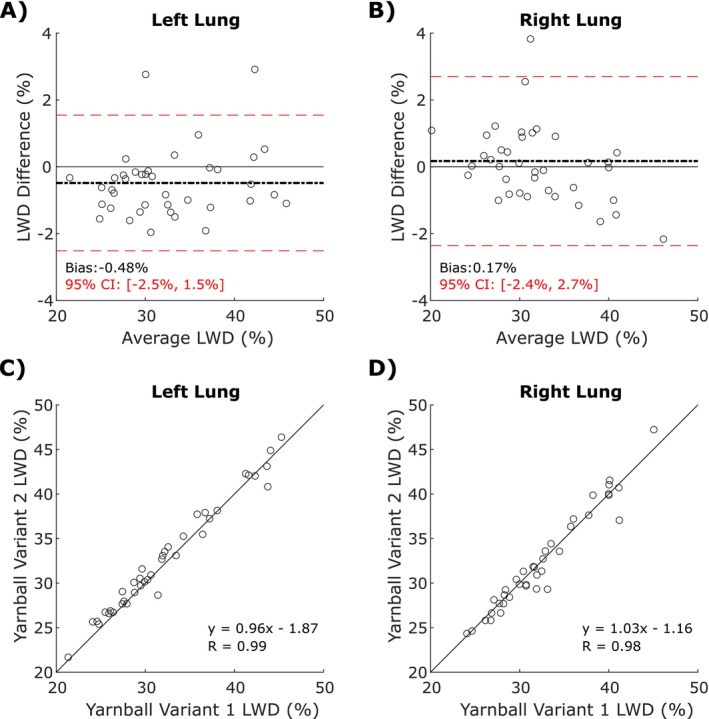
Dependence of lung water density on pulse sequence parameters: Bland–Altman plots (A, B) and the corresponding correlation plots (C, D) in the right and left lungs comparing the mean LWD in the subset of 39 COVID‐19 survivors imaged with both Yarnball pulse sequence variants. Biases (mean differences) are represented as a dotted black line, whereas the 95% confidence intervals (CI) are shown in red.

Analysis of LWD measurements in the 39 COVID‐19 survivors imaged with the two Yarnball pulse sequence variants showed an average difference of < 0.5% for LWD, 95% confidence intervals within ±3% LWD (Figure 6A,B). Correlation coefficients for the two Yarnball pulse sequences were greater than 0.98 in both lungs (Figure 6C,D).

## 4 Discussion

In this study, MRI‐derived water density of the lungs was evaluated as a novel quantitative measure of parenchyma tissue characteristics following recovery from COVID‐19 infection. Whole‐lung average LWD measured with MRI was shown to be significantly greater in the post‐COVID‐19 cohort compared with healthy controls. Association of elevated LWD with significantly elevated CRP and WCC measurements suggests a link between lung water and systemic inflammation, a noted clinical finding in COVID‐19 survivors [2]. This connection is further supported by the association of elevated LWD and acute COVID‐19 hospitalization, linking it to more severe acute infection and possibly post‐acute inflammation [30]. The positive correlation between FEV_1_/FVC and LWD suggests increased restriction with elevated LWD, possibly in relation to fibrosis.

However, the limited associations between elevated LWD and either exercise capacity (6MWD) or pulmonary function abnormalities suggest that elevated LWD is not a major contributor to long‐COVID‐19 symptoms. Interestingly, there were no differences in total lung water volume between the control and post‐COVID‐19 cohorts, although lung volumes in the post‐COVID‐19 group were smaller. This is likely related to the higher BMIs, which are also consistent with the observation of smaller but not clinically relevant FEV and FRC values in the post‐COVID‐19 cohort [31].

Normative LWD values in the healthy cohort in the current study were similar to previously reported values using the same UTE method [16, 22] and also with imaging studies performed at different field strengths and with acquisition approaches [15, 18, 21], approximately 27%–29% relative to reference tissues, with no reported sex differences. In addition, LWD values were shown to be insensitive to acquisition parameters, including changes in FOV, spatial resolution, and acquisition duration.

Increased LWD has been shown to be associated with elevated left ventricular filling pressures and BNP in heart failure [15], but given the normal BNP levels and lack of association between LWD and BNP in the post‐COVID‐19 cohort, the source of the elevated water density in the current study is likely not cardiogenic. The slightly reduced BNP values in those with elevated LWD likely reflect the higher BMI in this subgroup, as obesity is known to be associated with lower BNP values [32]. Similarly, the lack of relationship between elevated LWD and clinically important D‐dimer levels eliminates coagulopathy and its downstream effects [33] as a potential source of LWD elevation.

Baseline comorbidities such as obesity and hypertension have been linked to severe COVID‐19 infection and post‐acute outcomes [34, 35, 36]. In the current study, without an additional COVID‐negative control group with comparable comorbidities, the independent effects of these preexisting health issues and prior COVID‐19 infection cannot be determined. Indeed, comorbidities such as type 2 diabetes and obesity are associated with a proinflammatory state and more severe lung injury during acute COVID‐19 infection [36]. Diabetes and obesity are also linked to more severe post‐acute imaging and laboratory findings [37, 38]. Given that roughly 75% of participants with elevated LWD in the current study had one or more comorbidities, it is possible that the associated metabolic dysfunction predisposed these individuals to worse outcomes including lung damage and systemic inflammation. However, elevated mean LWD was also measured in post‐COVID‐19 participants without comorbidity, which suggests that prior COVID‐19 infection itself is an important contributing factor to lung water findings. The extent to which comorbidities contribute to post‐COVID‐19 lung water findings warrants future investigation.

Post‐COVID‐19 MRI studies to date have focused largely on functional aspects of the lung, with the use of Fourier decomposition [39, 40, 41] or inhaled gas contrast agents [42, 43, 44] most commonly reported in the literature, with a focus on ventilation. The proposed LWD metrics are likely complementary to these functional assessments and could be added to future functional lung imaging studies with minimal additional scan time. CT imaging is also sensitive to LWD and thus the findings of the current study are relevant for the more widely used CT imaging modality. However, future studies will be necessary to determine the relationship between MRI and CT‐derived LWD values.

LWD is directly modulated by lung inflation [22, 45] and thus the respiratory maneuvers used for image acquisition can have a large effect on LWD values. In the current study, all images were acquired during restful tidal breathing and reconstructed at FRC to ensure a consistent extent of lung inflation. LWD at FRC has been shown to increase dynamically following supine body positioning in association with an upward shift of the abdominal organs and diaphragm [22]. All lung images were acquired following a minimum of 20 min after patient positioning to minimize potential variability in lung inflation and thus LWD values.

Focal pathology including patchy regions of increased and decreased LWD was observed in the post‐COVID‐19 cohort. The current study did not include a quantitative analysis of the burden of these features, primarily due to the lack of robust methods to consistently identify and characterize these features in MRI. However, the illustrated LWD imaging approach was shown to clearly identify patchy pathology in the lung parenchyma, and quantitative analysis may be enabled by future studies focused on the development of novel postprocessing approaches.

## 5 Limitations

First, all participants in the post‐COVID‐19 cohort were infected with COVID‐19 and imaged prior to the outbreak of the omicron SARS‐CoV‐2 variant in late 2021. The omicron variant has been observed to have milder outcomes and radiographic findings [46]; therefore, it is unclear if the lung water findings of the current study are applicable in populations infected with Omicron and subsequent variants. Second, it is possible that individuals in the healthy cohort, who self‐reported no previous COVID‐19 infection, had an unknown symptom‐free COVID‐19 infection. Third, the normalization technique used in this study to calculate LWD images presumes that the solid tissues surrounding the lung cavity have consistent and uniform water density; however, this assumption may be violated by the presence of increasing volumes of fat in the reference tissue regions. As fat has a proton density similar to pure water and thus higher than tissue proton density [47], the presence of fat in the reference tissue will increase the reference signal intensity used in the normalization process, causing a potential systematic underestimation of LWD. This is noteworthy as the post‐COVID‐19 cohort examined in this study had a significantly greater BMI than the healthy controls and commonly presented with a greater fat content in reference tissue regions. Although the full effect of fat inclusion in reference tissue has yet to be characterized, it is possible that LWD in the post‐COVID‐19 cohort of this study was underestimated. Underestimation may also arise from pathologically increased water content in the reference tissues, as may be the case in conditions such as heart failure and chronic kidney disease. Finally, the presence of co‐morbidities in the post‐COVID‐19 cohort complicates findings of elevated lung water in COVID‐19 survivors. Without an additional COVID‐negative control group with comparable comorbidities, conclusions about the independent effects of these pre‐existing health issues and prior COVID‐19 infection cannot be drawn.

### 5.1 Conclusions

This cross‐sectional study compared MRI‐derived LWD in COVID‐19 survivors and healthy controls. Despite exhibiting minimal functional impairment, post‐COVID‐19 participants were found to have higher mean LWD in both lungs, independent of sex and age. The post‐COVID‐19 participants with elevated LWD had higher concentrations of inflammatory plasma biomarkers, higher rates of hospitalization during the COVID‐19 infection, and a greater incidence of comorbidities such as obesity and diabetes.
